# Nomothetic and idiographic determinants of dietary lapses and adherence: an exploratory mixed methods ecological momentary assessment study of open-text responses

**DOI:** 10.3389/fnut.2026.1869531

**Published:** 2026-08-14

**Authors:** Hannah van Alebeek, Anna Schröder, Dennis John, Thomas Probst

**Affiliations:** 1 Department of Psychology, University of Salzburg, Salzburg, Austria; 2 Institute for Applied Research and Evaluation, Lutheran University of Applied Sciences, Nuremberg, Germany

**Keywords:** dietary adherence, dietary lapses, eating, ecological momentary assessment, mixed method

## Abstract

**Objective:**

Dietary interventions often fail to achieve sustained behavior change, partly due to limited understanding of momentary factors driving dietary lapses and instances of goal adherence. Given constrained item pools in ecological momentary assessment (EMA), it remains unclear which factors are most relevant to individuals in daily life. This study therefore examined dieters’ self-reported momentary reasons for lapses and adherence and whether these are broadly applicable (nomothetic) or individual-specific (idiographic).

**Methods:**

Data were drawn from two EMA studies (*N* = 199) in which participants reported lapses and adherence every 3.5 h (over 1 or 2 weeks) and provided open-text explanations. In total, 5,402 reasons were analyzed using AI-assisted qualitative content analysis. Categories were evaluated with three indices to assess their nomothetic versus idiographic relevance.

**Results:**

Overarching categories were similar for lapses and adherence (self-regulation, food availability, social influences, lack of opportunity, unplanned circumstances, emotional, physiological, craving-related factors), but subcategories differed (e.g., deliberate exceptions vs. routines and strategies). No lapse category was reported by >47.73% of participants, indicating substantial idiographic variability. The most broadly applicable factors across participants were cravings, physiological factors (especially hunger), and social influences for lapses, and lack of opportunity (especially being occupied) for goal adherence.

**Conclusion:**

Participants highlighted (sub)categories typically underrepresented in lapse prediction models (e.g., health-related states, availability of goal-congruent foods, lack of opportunity). Substantial individual variability further suggests that lapse reasons do not generalize across participants and that personalized item selection would probably improve lapse prediction and just-in-time adaptive interventions.

## 1 Introduction

Attaining a healthy diet has been recognized as one of the most important lifestyle factors in the prevention of various diseases such as diabetes, cardiovascular issues, and musculoskeletal problems (1). Consequently, numerous dietary interventions have been developed to support behavioral change, and around 60% of Austrians and Germans intent to lose weight or change their diet [i.e., eat less unhealthy food types (2)]. However, meta-analyses show that such efforts are often unsuccessful, with limited long-term maintenance of results (3, 4).

To better understand related barriers, research has examined psychological determinants of dietary non-adherence, including dichotomous thinking, low inhibitory control, and primary dieting motives such as weight loss or appearance (5). Further, with the rise of smartphone-based studies which enable the assessment of temporally fluctuating psychological and environmental states in daily life [i.e., ecological momentary assessment, EMA (6)], research has also identified momentary predictors for instances of lapses or overeating such as current food cravings, availability or social cues (7). In EMA research, a “lapse” is typically defined as any instance of non-adherence to intended healthy eating since the last report (8). Although single lapses may not be inherently detrimental, they are assumed to undermine long-term adherence by for example reducing self-efficacy and increasing the risk of a full relapse [i.e., return to baseline eating patterns (9)]. Accordingly, recent studies have applied machine learning models to EMA data to forecast lapse risk in real time (10, 11), with the final goal to intervene right before and prevent lapse occurrence [just-in-time adaptive interventions; e.g., see (12)].

Despite progress in identifying momentary predictors of dietary lapses, it remains unclear which of these dynamic factors are most relevant for a specific individual. This problem is particularly evident in EMA research, where item pools are limited to minimize burden of repeated assessments. Currently, only about half of EMA studies have grounded their item selection in explicit theoretical frameworks (8). Moreover, employed theories are often developed at the between-person level (i.e., who is prone to diet non-adherence), and thus it remains uncertain how much relevance identified factors have for within-person processes (i.e., when a lapse occurs). However, even within theories designed for within-person dynamics, item selection may remain challenging if reasons for (non-)adherence are highly idiographic [as for example shown for binge eating prediction (13)]. For example, factors such as food cravings but not time pressure may reliably predict lapses occurrence in some individuals, while for others time pressure may be the most relevant. Such potential variability raises the question of whether a single (theoretically derived) nomothetic item pool can adequately forecast lapse occurrence, which would be however crucial for the effectiveness of personalized just-in-time adaptive interventions (13).

One way to examine which factors are most relevant for dietary lapse prediction across or within individuals is to directly ask dieters themselves. While studies have qualitatively investigated reasons for more long-term diet failures (5) or lapse instances (14) using focus groups or interviews, no study has systematically investigated which factors dieters perceive as most important for instances of dietary lapses or time windows of no lapses (termed dietary adherence in the following) directly after their occurrence in their daily lives. Such an approach may confirm whether currently employed factors are also recognized as relevant by dieters themselves, while also highlighting influences that may have been overlooked. Moreover, repeated within-person assessments can help to explore factors that are commonly reported across individuals and factors that, while less prevalent at the group level, recur consistently within specific individuals. This distinction can inform whether future models for momentary lapse prediction can rely on fixed nomothetic predictors or should shift towards to more idiographic item selection.

To our knowledge, no mixed-methods study has systematically examined dieters’ momentary self-reported reasons for lapses and adherence within an EMA framework. To address this gap, we applied a sequential exploratory mixed-methods design in which qualitative analyses were conducted first to identify relevant categories, followed by quantitative analyses examining their prevalence (15). The qualitative analyses were primarily inductive, deriving categories directly from participants’ responses, with theory applied only at the final step to structure the resulting, data-driven category system. Specifically, we first analyzed qualitative responses from two EMA studies in which participants repeatedly reported whether they had experienced a dietary lapse within the past 3.5 h and indicated the reasons they considered most important for the outcome of their recent dietary goal pursuit (i.e., lapse or adherence). After identification of relevant factor categories, we specifically examined which factors were commonly reported across individuals (i.e., suitable for nomothetic lapse prediction) and which were more specific to certain individuals (i.e., suitable for idiographic lapse prediction). Thereby, we aim to provide a “basic set” of reasons with relevance to most participants which may be combined with an “optional set” of individually-selected reasons in future research.

To summarize, the following research questions (RQ) are addressed:

RQ1: Which factors do participants identify as relevant for their lapses and adherence in daily life?

RQ2: How frequently are the factors reported across and within individuals?

## 2 Methods

Data for the current analyses stem from two EMA studies and can be found on OSF.^1^ Study 1 comprised 2 weeks of baseline assessment followed by one intervention week, while Study 2 lasted 1 week and consisted of two arms (control and intervention). We included the baseline data from Study 1 and the control-arm data from Study 2. As both studies employed the same EMA item pool and sampling scheme, the main differences were the sampling duration of included data (2 vs. 1 week). A-priori hypotheses for these studies were pre-registered (Study 1: https://osf.io/ugx26; Study 2: https://osf.io/mta53) but current qualitative analyses were not. Ethical approval for both studies was granted by the ethics committee of the University of Salzburg (GZ 56/2024).

### 2.1 Participants

In both studies, participants were recruited via social media posts and university course announcements in Austria and Germany. They were required to set a goal at study start that they intended to pursue for the duration of the study. Participants who declined to set such a goal were not eligible to proceed with the study. Other exclusion criteria in both studies included being under 18 years of age or lacking sufficient German language proficiency to understand the study instructions.

### 2.2 Procedure

#### 2.2.1 Pre assessment

At study start, participants provided demographic information, including age, gender, nationality, height, weight, education level, dietary preference (e.g., vegan, vegetarian, pescetarian, omnivore, other), and history of eating disorders. Participants were then instructed to set a diet-related goal to be pursued for the duration of the study (see all reported goals in Supplement A). To increase comparability between participants, the goal was required to involve some form of intake reduction (e.g., eating less in general or less of a certain food). They then completed several trait questionnaires not relevant for the current analyses (see Supplement B). In Study 1, participants then entered the EMA phase. In Study 2, participants were randomized to either an intervention condition (temporal distancing with if-then planning), or a no-intervention control condition before entering the EMA phase.

#### 2.2.2 EMA-phase

In Study 1, the EMA phase lasted 14 days, whereas in Study 2 it lasted 7 days. During this period, participants received four time-contingent questionnaires per day (at 11:00, 14:30, 18:00, and 21:30). App push notifications prompted participants to report (a) the availability of foods incongruent with their dietary goal (0 = ‘not at all’ to 100 = ‘all the time’), (b) consumption of these foods (0 = ‘nothing’ to 100 = ‘very much’) and (c) adherence to their dietary goal since the previous prompt (i.e., over the past 3.5 h; ‘yes’ or ‘no’). Depending on their recent goal adherence (coded as lapse or adherence), they were asked for possible reasons using an open-text response with the following instructions: “Please state the reasons why you were [not] able to adhere to your dietary goal for the last 3.5 h (the most important one first):”. In sum, 5,190 text responses on this item were used for qualitative analyses in the current study. Following that, other items not relevant to the context of the current study, namely attributions (3 items), emotions (5 items), self-efficacy (1 item), and intended effort (1 item) were assessed. EMA items did not differ between both studies.

After the EMA phase, participants in Study 1 entered a one-week attributional retraining intervention before study completion, whereas participants in Study 2 completed a manipulation check and then ended the study.

### 2.3 Data analyses

#### 2.3.1 RQ 1: qualitative content analysis

##### 2.3.1.1 Qualitative analyses procedure

Qualitative data, i.e., text responses were analyzed using ‘Conversational Analysis to the power of AI (CA^AI^)’ (16). Unlike traditional categorical coding schemes used in qualitative content analysis (17), CA^AI^ identifies themes emerging from the data through a dialogic process between researchers and Artificial Intelligence (AI). We used the software QInsights to conduct CA^AI^ and analyze the EMA text responses. Specifically, CA^AI^ was applied to identify themes directly from the qualitative data on reasons for dietary adherence and lapses. QInsights complies with GDPR standards, and only anonymized text responses were imported into the software. Moreover, data analysis within QInsights is based on a Retrieval-Augmented Generation (RAG) system, meaning that the AI’s output is generated exclusively from the provided dataset rather than from external training data. The set of text responses was processed using the following prompt in QInsights^2^:

*“Which themes are mentioned in the reasons for lapses and adherence to dietary goals? Separate the themes according to lapses and adherence. List 3–8 themes separately for lapses as well as adherence, depending on the number identified. The number of themes for lapses and adherence need not to be equal. For each identified theme, provide a brief explanatory text. Include two examples from the data for each theme.”*

Because QInsights processes only a limited number of responses at a time (approximately 1,000) while preserving inductive specificity—larger inputs yield less differentiated themes—the text responses were analyzed in five batches of approximately 1,000 responses each, applying the above prompt separately to each batch. This procedure resulted in five separate theme lists. Across these lists (Supplement C1), recurring themes included self-regulation, emotional factors, physiological factors, cravings/appetite, food (un)availability, (lack of) opportunities, and social factors, which were subsequently consolidated into the category system presented in Supplement C2.

##### 2.3.1.2 Development of coding manual

To develop the coding manual, we used a GDPR-compliant GPT-based chatbot provided by the Lutheran University of Applied Sciences in Nuremberg to consolidate the five theme lists into a unified category system. This procedure was conducted separately for lapses and adherence (i.e., two runs) to maximize the conceptual clarity and discriminative power of the resulting categories. The following prompt was issued:

*“Here are five lists containing themes on adherence [or lapses] to dietary goals. Create out of them an overall category system for use in qualitative content analysis, ensuring that all themes from the five lists are fully represented and that categories are clearly distinguishable. Provide a brief explanatory text for each category. Include five text examples from the data for each category. The five theme lists are: [list of themes].”*

The first and second authors reviewed and compared the two separate category systems generated by the GPT-based chatbot. They merged overlapping categories, expanded categories where necessary, and introduced subcategories based on the extracted themes, following established procedures for developing coding manuals in qualitative content analysis. The coding manual was thus developed primarily inductively: theoretical knowledge, namely an attributional perspective distinguishing internal versus external reasons, was applied only at this final step to structure the otherwise data-driven categories. As a result, the category systems for adherence and lapses were largely analogous in structure. The first version of the coding manual therefore comprised parallel main categories for adherence and lapses. Before the final coding, the AI-generated output had undergone two rounds of communicative validation by the first and second authors, first the QInsights theme lists, then the chatbot-generated category systems.

To evaluate and refine the coding system, approximately 30% of the data were independently coded by the first and second authors using this initial version of the manual. This phase served as a pilot test to iteratively validate and further specify category definitions and coding rules. The final coding manual comprised a structured set of categories for lapses and adherence, a comprehensive overview table including all main and subcategories, detailed category definitions, example responses for each subcategory, and explicit coding instructions. The coding manual in its original language (German) is available in Supplement C2 and summarized in the Results Section.

##### 2.3.1.3 Interrater reliability for coding system

Two trained student assistants served as independent coders and were instructed using the coding manual. The coding procedure comprised two steps. In step 1, coders determined the number of coding units per response. Interrater reliability for this decision was assessed using Cohen’s *κ*. Agreement was moderate according to the benchmarks proposed by Landis and Koch (29), *κ* = 0.47, 95% CI [0.43, 0.52], with 391 disagreements out of 5,190 text responses. Although *κ* = 0.47 indicates moderate agreement, segmentation decisions in qualitative content analysis are inherently interpretative and typically show lower reliability than subsequent category assignment (18). Discrepancies were resolved by the first author, who reviewed all conflicts and made the final decision regarding the number of coding units per response. In total, 5,697 units were derived from the initial 5,190 text responses.

In step 2, the finalized coding units were independently assigned to categories according to the coding manual. Interrater reliability for category assignment was high, *κ* = 0.88, 95% CI [0.86, 0.89], indicating almost perfect agreement (29), with only 721 disagreements. Discrepancies were discussed between the coders, and final decisions were reviewed and approved by the first author to ensure unambiguous categorization of each coding unit.^3^

#### 2.3.2 RQ 2: quantitative analyses

Of the 5,697 coding units identified during qualitative coding (based on the 5,190 text responses), 5,402 were retained for quantitative analyses (based on 4,900 text responses). This is because two participants were identified who took part in both studies after the categorization process, and their data were excluded from the second study to avoid dependency (54 codings). Additionally, 241 coding units (55 lapse and 186 adherence entries) were excluded because they were categorized as “No reasons given.” These responses either described behavior without providing a reason (e.g., “ate only ice cream,” “ate an apple,” “had fruit for breakfast”) or explicitly indicated the absence of a reason (e.g., “no particular reason”). Importantly, no responses were coded as “Other,” suggesting that the categorization system was sufficiently comprehensive for coders to assign all reported reasons to an existing category.

##### 2.3.2.1 Predicted probability of reported reasons across and within individuals

We investigated which categories would be sui– candidates for nomothetic lapse prediction (i.e., reported by most individuals with relatively high and homogeneous probability) versus those better suited as idiographic predictors (i.e., not reported by everyone, but when reported, occurring with relatively high probability within that individual). To evaluate this, we used four complementary indices: (1) Average User Prevalence: population-averaged probability of reporting a given category, (2) User Variance: the between-person heterogeneity in predicted probabilities, (3) Top-User Probability: the predicted probability among high-use individuals, operationalized as the 90th percentile of the participant-specific predicted probabilities, and (4) User Coverage: the proportion of individuals who reported a given category at least once. The former three indices were derived from separate hierarchical logistic regression models that leverage partial pooling to account for unequal numbers of observations across participants (e.g., due to differences in compliance and/or sampling duration across studies), thereby avoiding bias toward individuals contributing more data, as would occur in raw frequency-based estimates (raw frequencies are displayed in Supplement D). Specifically, we predicted the occurrence vs. non-occurrence (coded as 1 vs. 0) of each category with a fixed intercept as well as a random intercept per participant, as shown in Equation 1.


$$\text{logit}(P(Y_{\mathrm{itc}}=1))=\beta_{0}+u_{i},$$ (1)

With $Y_{\mathrm{itc}}$ indicating whether an individual i reported category c at time t, and $u_{i}∼N(0,\sigma_{u}^{2})\;$ represents the participant-specific random intercept.

Ad index (1) The Average User Prevalence was quantified using the marginal effect of the intercepts. Higher values indicate a higher overall probability of category reporting, independent of participant-specific deviations. Ad index (2) The User Variance (i.e., between-person heterogeneity as depicted in Supplement E) was quantified using the intraclass correlation coefficient (ICC), which reflects the proportion of variance attributable to differences between participants. Lower ICCs indicate relatively homogeneous reporting across individuals, whereas higher ICCs indicate substantial between-participant heterogeneity. Ad index (3) The Top-User Probability was defined as the 90th percentile of the distribution of participant-specific predicted probability for a given category. This metric reflects the expected probability of category occurrence among individuals who use that category most frequently and thus captures the degree of within-person recurrence among high-use participants. Ad index (4) User Coverage is not model-based and therefore sensitive to sampling differences. It however provides a descriptive measure of the proportion of individuals for whom the category was identified as relevant at least once, while the remaining proportion reflects those for whom it was never stated as relevant during the observation period. These indices were calculated separately for lapses and adherence for mid- and low-level categories. As mid-level categories were largely analogous between adherence and lapses, we additionally calculated the indices for lapses and adherence combined (displayed in Supplement D).

Categories with highest Average User Prevalence and lowest User Variance (relative to other categories) were considered most suitable as nomothetic predictors, as the corresponding EMA items are likely relevant for most individuals and operate similarly across them. In contrast, categories with highest User Variance and highest Top-User Probability (relative to other categories) were considered most suitable as idiographic predictors, as the corresponding EMA items are not widely endorsed across the sample but are strongly concentrated within specific subsets of individuals. Categories that do not clearly meet either profile (i.e., do not show most extreme values in User Variance together with highest values in Average User Prevalence or Top-User Probability) were not selected as nomothetic or idiographic candidates.

## 3 Results

### 3.1 Sample characteristics

The final sample included 4,900 textboxes from 199 participants. Each textbox could contain several coding units, resulting in 5,402 distinct reasons for recent lapses or adherence. Participants were predominantly female (79.72%), had a mean age of 29.81 (SD = 13.83, min = 18, max = 76) and a BMI of 23.72 (SD = 4.72, min = 15.59, max = 42.67). They reported a lapse in 20.69% of their entries and thus, analyses of lapses were based on 1,014 textboxes, whereas analyses of adherence were based on 3,886 textboxes. Differences in sample characteristics between Study 1 and Study 2 can be found in the Supplement D (Table D4).

### 3.2 RQ1: which factors do participants identify as relevant for lapses and adherence in daily life?

Participants described both internal and external factors as influencing their dietary goal attainment, representing the highest-level categories. Internal reasons referred to distinct processes within the individual whereas external factors reflected situational and social influences that either facilitated or hindered adherence. Within both domains, we identified several mid-level categories that were largely comparable across adherence and lapses (see below). However, certain mid-level categories were described in greater depth (i.e., lowest level) depending on whether participants were providing reasons for lapses or adherence. This highlights that the same broad factors manifest differently in contexts of adherence versus lapses:

#### 3.2.1 Internal reasons for lapses

For lapses, participants reported several distinct internal reasons related to a failure in *self-regulation.* Subcategories included (1) lack of self-control such as “poor impulse control today,” (2) low motivation such as “little motivation to think about the goal,” (3) poor planning such as “no packed lunch,” and (4) deliberate exceptions such as “I’m not tracking today because we wanted to order food and I need a break from tracking.”

*Physiological* and *emotional factors* were also identified as contributing to lapses. Frequently reported physiological factors included (1) health-related and (2) sleep-related factors that impaired the ability to adhere to goals (e.g., “Premenstrual Syndrome,” or “Tired, so pasta for dinner”), as well as (3) high hunger levels (e.g., “very hungry” or “Haven’t eaten in a long time”). Regarding emotional factors, participants commonly reported (1) negative emotion in general such as “frustration” or “boredom,” (2) stress specifically, and (3) the use of eating to regulate negative states (e.g., “Comfort Eating” or “Plundering the candy supply to feel better”). Finally, *cravings*, defined as a subjective experience of desire, appetite, and strong urges for food (directed either at specific foods or food in general; e.g., “I was craving something sweet”), were also reported as a reason for lapses, but no further subcategorization emerged. Cravings are thus distinct from hunger in that they reflect a subjective desire rather than a physiological need.

#### 3.2.2 External reasons for lapses

External lapse reasons were related to food availability, social factors, unplanned circumstances and lack of opportunities. Regarding *food availability*, lapses were attributed either to (1) easy availability of goal-incongruent foods, e.g., “Candy within reach” or (2) no availability of goal-congruent alternatives, e.g., “There was no vegetarian option at work.” *Social factors* included the involvement of third parties such as social gatherings, peer pressure, or expectations from others that influenced eating behavior. *Unplanned circumstances* included all factors beyond the individual’s control, such as being unaware of specific ingredients in foods or that the expiration date would have passed otherwise, and *lack of opportunities* to adhere to goals included time or situational constraints such that participants were too busy to cook something goal-congruent or being on the move. For the latter three categories, no further subcategorization emerged.

#### 3.2.3 Internal reasons for adherence

For adherence, participants reported several distinct internal reasons related to *self-regulation* and *physiological factors*. Frequent mentions for self-regulation were (1) strong self-control such as “iron will,” or “control,” (2) high motivation such as “wanting to be healthy” or “I am still motivated,” (3) good planning and preparation such as “I brought my own protein-rich snack with me” or “already made sure not to buy any wheat products when shopping,” (4) established routines such as “it has become a habit” or “I have reestablished a routine,” and (5) specific strategies such as “16–8 diet” or “Gave all ‘dangerous’ foods to others.” Frequently reported p*hysiological factors* included (1) health-related factors such that participants did not eat anything due to stomach pain or nausea, (2) sleep-related factors such as “too tired to eat,” and (3) low hunger levels such as “I’m full from lunch” or “no hunger.”

*Emotional factors* were also reported, with participants for example describing the absence of destabilizing states (e.g., “I was not bored”), or the presence of self-evaluative emotions due to past lapses (e.g., “bad conscience”). *Craving*, defined in the same way as for lapses, was stated as an important reason for goal adherence, typically in terms of low levels of craving (e.g., “No desire for it”). For emotional and craving-related factors, no meaningful further subcategorization emerged.

#### 3.2.4 External reasons for adherence

External reasons for adherence related (as for lapses) to food availability, social factors, and lack of opportunity, but not to unplanned circumstances. Regarding food availability, adherence was attributed either to (1) no availability of goal-incongruent foods (e.g., “There wasn’t anything ‘unreasonable’ to eat”) or (2) the easy availability of goal-congruent alternatives (e.g., “A good selection of cafeteria food available”). *Social factors* included motivation or support from others as well as motivation derived from study participation; no further subcategorization emerged. Reasons related to a *lack of opportunity* to violate dietary goals could be further differentiated into several distinct external constraints: (1) being occupied, such that participants were distracted or had no time to eat, (2) being on the move, which prevented eating (3) sleeping, during which eating was not possible, and (4) other reasons such as already having brushed their teeth.

### 3.3 RQ 2: how frequently are factors reported across and within individuals?

#### 3.3.1 Nomothetic and idiographic structure of lapse-related reasons

As shown in Table 1, no mid- or lower-level category was endorsed at least once by more than half of the participants (i.e., User Coverage ≤ 47.73). This indicates that none of the lapse reasons were universally reported across the sample, suggesting limited generalizability at the population level.

**Table 1:** Reasons for lapses by higher- and lower-level categories.

Category			Indices			
High-level	Mid-level	Lower-level	Average User Prevalence	User Variance	Top-User Probability	User Coverage (≥1 occurrence)
Internal	Lack of self-regulation	No self-control	0.68%	57.95%	6.24%	13.63%
		No motivation	0.01%	95.45%	<0.01%	7.39%
		No planning	0.81%	NA	0.81%	5.11%
		Deliberate exception	0.57%	72.12%	11.59%	18.75%
		*Overall*	6.79%	34.90%	18.66%	38.64%
	Emotional factors	Negative emotions	2.52%	47.45%	11.45%	23.86%
		Stress	0.10%	88.69%	15.96%	18.75%
		Emotional support	0.03%	86.99%	0.03%	6.82%
		*Overall*	6.71%	47.96%	34.04%	38.64%
	Physiological factors	Health-related	0.03%	91.43%	10.57%	12.50%
		Sleep-related	0.05%	88.36%	2.07%	10.23%
		Hunger	2.81%	46.16%	13.11%	23.86%
		*Overall*	9.15%	32.80%	25.11%	38.07%
	Cravings		11.83%	24.14%	24.25%	47.73%
External	(Un)availability	Goal-incongruent foods	4.10%	47.32%	20.01%	31.25%
		Alternatives	0.05%	89.75%	12.47%	14.21%
		*Overall*	7.68%	39.49%	28.05%	41.48%
	Lack of opportunities		3.66%	52.69%	30.53%	28.98%
	Social factors		9.03%	29.99%	24.98%	40.91%
	Unplanned circumstances		1.79%	42.12%	7.89%	18.75%

NA indicates that the database was too small to estimate the intraclass correlation coefficient, used as an index for User Variance.

**Figure 1:**
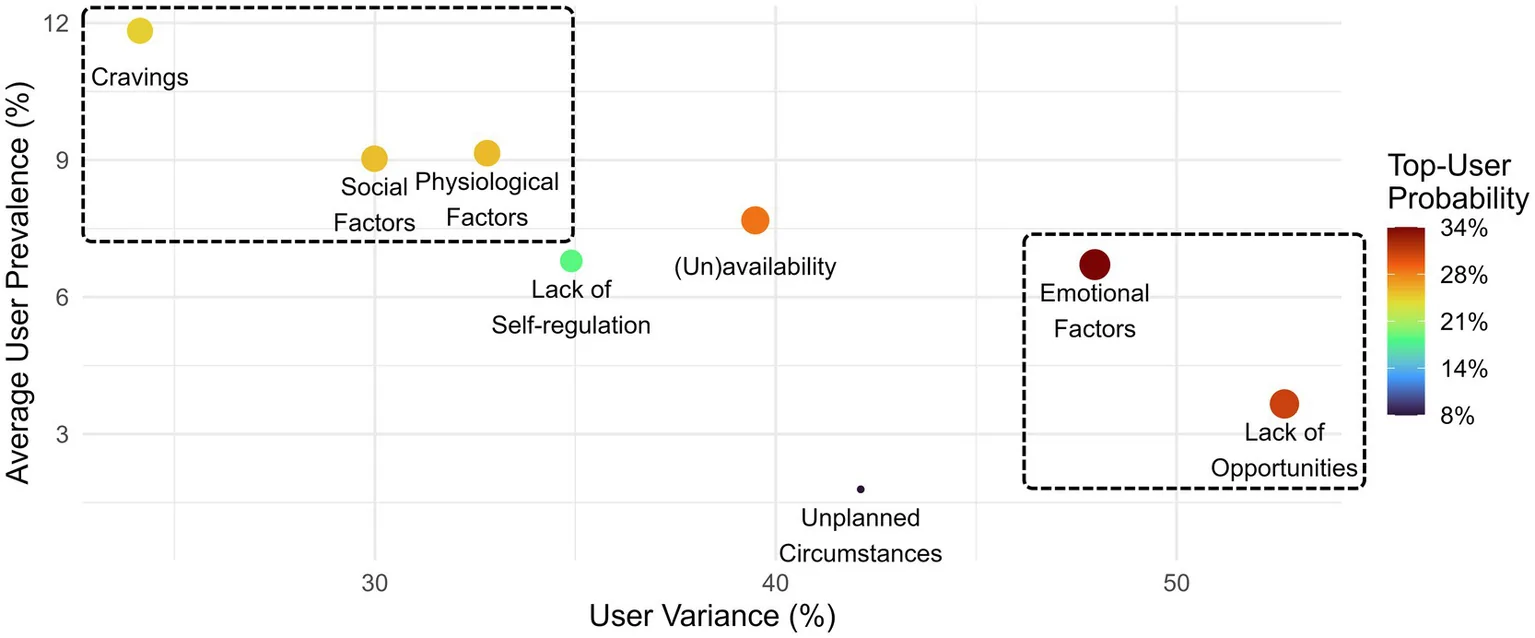
Nomothetic and idiographic structure of mid-level lapse categories. Boxes indicate categories considered more suitable than other categories as either nomothetic (i.e., highest Average User Prevalence and lowest User Variance) or idiographic (i.e., highest User Variance and highest Top-User Probability) lapse predictors.

However, mid-level categories with the highest population-averaged probability (i.e., Average User Prevalence) were cravings (11.83%), physiological factors (9.15%) and social factors (9.03%), indicating most frequent reporting across individuals. Notably, all three also exhibited lowest between-person heterogeneity (i.e., User Variance ≤ 32.80%), indicating relatively consistent reporting across individuals. Within physiological factors, which comprised three lower-level subcategories, hunger showed the highest Average User Prevalence and the lowest User Variance. The combination of highest Average User Prevalence and lowest User Variance (see upper-left box in Figure 1) suggests that these mid-level categories, and specifically hunger within physiological factors, would be most suitability as nomothetic lapse predictors.

In contrast, emotional factors and lack of opportunities to adhere to dietary goals appear particularly relevant as idiographic predictors. These categories exhibited highest between-person heterogeneity in reporting probabilities (i.e., User Variance ≥ 47.96%), and individuals who reported them did so frequently (i.e., Top-User Probabilities was highest exceeding ≥ 30.53%). Within emotional factors, which comprised three lower-level subcategories, stress had highest User Variance and Top-User Probability. This pattern, displayed in the lower-right box of Figure 1, suggests that these mid-level categories (and specifically stress within emotional factors) are strongly concentrated within a subset of individuals, indicating that they may represent especially promising candidates for lapse prediction at the individual level (i.e., idiographic predictors).

#### 3.3.2 Nomothetic and idiograohic structure of adherence-related reasons

As shown in Table 2, self-regulation-related factors and lack of opportunities to violate dietary goals were reported at least once by more than 80% of participants (i.e., User Coverage ≥ 80.10%). This indicates that these categories were relatively widely endorsed across the sample. All other mid- and lower-level categories were reported by a maximum of 55.61% of participants at least once, indicating substantially lower coverage compared to self-regulation-related factors and lack of opportunities.

**Table 2:** Reasons for adherence by higher- and lower-level categories.

Category				Indices		
High-level	Mid-level	Lower-level	Average User Prevalence	User Variance	Top-User Probability	User Coverage (≥1 occurrence)
Internal	Self-regulation	Self-control	3.57%	54.33%	31.46%	49.49%
		Motivation	1.80%	65.38%	26.87%	41.84%
		Planning	0.72%	70.61%	19.56%	32.14%
		Routines	0.04%	86.58%	6.39%	18.88%
		Strategies	0.01%	90.66%	2.69%	13.27%
		*Overall*	25.73%	47.99%	74.41%	80.61%
	Emotional factors		0.08%	77.92%	2.77%	15.82%
	Physiological factors	Health-related	0.03%	83.82%	1.73%	12.25%
		Sleep-related	0.01%	84.64%	0.01%	7.14%
		Hunger	2.18%	49.40%	15.12%	38.78%
		*Overall*	3.78%	41.33%	17.28%	46.94%
	No cravings		2.76%	56.16%	26.55%	43.88%
External	(Un)availability	Goal-Incongruent Foods	2.37%	47.64%	17.03%	31.25%
		Alternatives	0.53%	69.69%	15.49%	14.21%
		*Overall*	5.41%	45.47%	28.77%	55.61%
	Lack of opportunities	Occupied	9.35%	39.31%	34.49%	66.84%
		On the Way	4.61%	41.94%	22.41%	47.96%
		Sleeping	1.15%	45.79%	7.52%	28.57%
		Other Factors	0.43%	65.91%	9.11%	23.98%
		*Overall*	26.78%	35.07%	60.13%	80.10%
	Social factors		0.76%	50.37%	5.43%	25.00%

**Figure 2:**
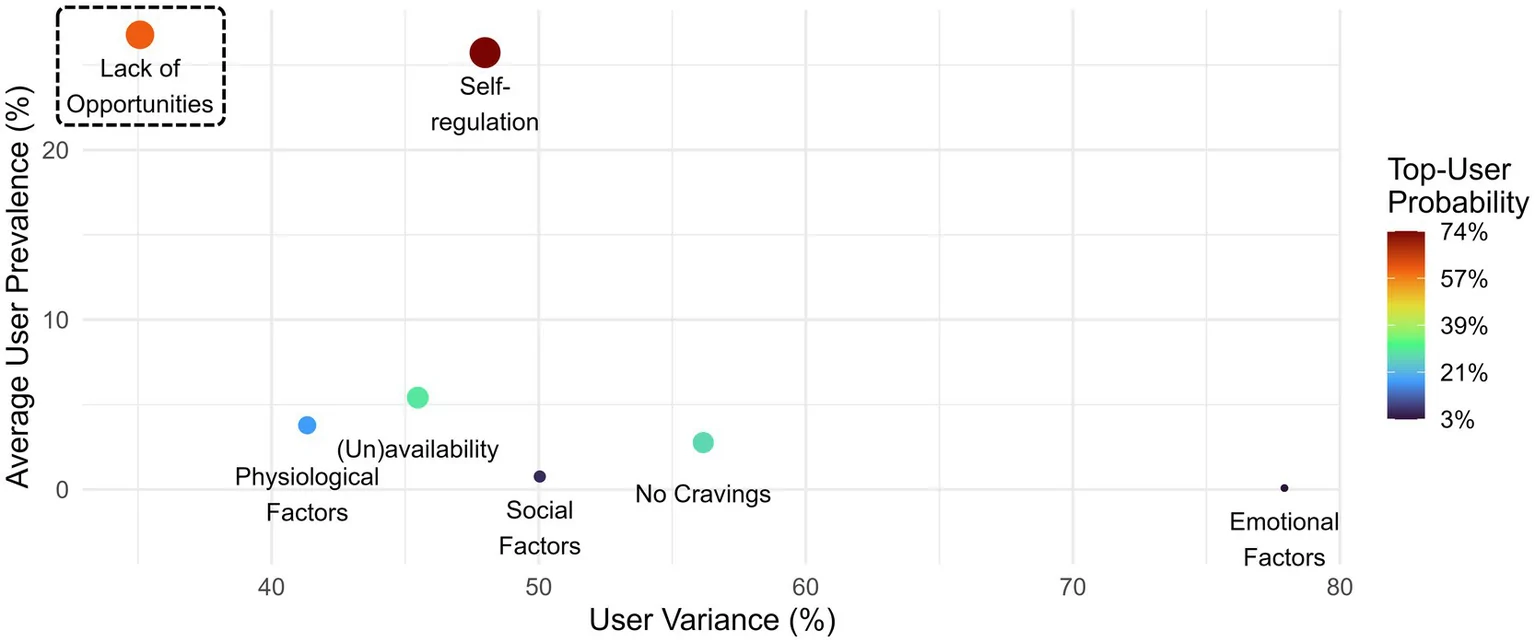
Nomothetic and idiographic structure of mid-level adherence categories. The box indicates the category considered more suitable than other categories as nomothetic (i.e., highest Average User Prevalence and lowest User Variance) dietary adherence predictors. No category stood out as a clear idiographic predictor (i.e., highest User Variance and highest Top-User Probability).

Self-regulation-related factors and lack of opportunities also stood out among mid-level categories in terms of highest Average User Prevalence; both exceeded 25%, whereas all other categories remained below approximately 5.5% (see Table 2). Notable, lack of opportunities also exhibited the lowest User Variance across all mid-level categories (see Figure 2). This pattern of highest reporting probability across individuals and lowest between-person heterogeneity suggests that lack of opportunities would be most suitable as nomothetic predictors for instances of dietary adherence. Within lack of opportunities, being occupied showed the highest Average User Prevalence and the lowest User Variance.

No category clearly stood out as being suitable as an idiographic predictor for adherence (i.e., highest User Variance and highest Top-User Probability).

## 4 Discussion

Using self-reports collected directly after dietary lapses or adherence, this mixed-methods study identified factors dieters themselves perceive as relevant for dietary goal pursuit in their daily life. This is particularly important as item pools are relatively restricted in EMA studies and there is no clear (theoretical) guidance on the most informative lapse predictors. To address this, we combined qualitative content analyses of reported reasons (RQ1) with quantitative analyses of between- and within-person level reporting frequency (RQ2). In the following, we first compare the qualitatively identified factors with established lapse predictors in prior EMA studies and then evaluate their generalizability across individuals to inform their suitability in nomothetic item pools.

### 4.1 Overlap between identified reasons and commonly used EMA predictors

As a reference, we draw on a recent machine learning-assisted systematic review and meta-analysis that identified 213 unique lapse predictors across 87 studies (8). In contrast to prior EMA research, which predominantly focused on internal predictors [i.e., only around 17% of predictors reflect social and environmental factors in (8)], participants in the current study reported a relatively balanced distribution of internal and external reasons. Accordingly, dieters perceive their behavior as more contextually embedded than is typically reflected in EMA research. This may be because many mechanistic studies on dietary violations have been conducted in the lab (19), where external influences are held constant, potentially leading to an underrepresentation of external factors in the literature and consequently, in EMA studies as well [as highlighted by (20)]. Current findings, however, suggest that incorporating external factors would probably improve explanatory validity of models predicting dietary goal pursuit in daily life.

At mid-level categories, most participant-reported factors have been employed in prior EMA research, including factors related to self-regulation, affect, physiological states, desire, food availability, opportunities, and social context (but not unplanned circumstances). However, several discrepancies emerge at a more fine-grained level. Physical states in prior EMA research typically included hunger and sleep-related factors, whereas participants in the current study additionally reported health-related factors such as PMS, nausea or hangovers [as also identified in another qualitative study analyzing responses from a semi-structured interviews (21)]. Similarly, availability has typically been assessed for goal-incongruent foods or general food availability, while participants also emphasized the availability of goal-congruent alternatives. Lack of opportunity was also more broadly captured in participants’ reports. Previous studies typically focused on participants’ location (e.g., being at school or at work), whereas current participants specifically highlighted a lack of opportunity due to being occupied, being on the way, or simply sleeping within the time window before the first prompt was sent at 11:30, which need to be considered depending on the chosen sampling scheme.

Another notable discrepancy concerns emotional factors. While prior studies often included negative and positive emotions as lapse predictors, only negative emotional states emerged as a distinct category in current participants’ reports. This discrepancy is unlikely due to biased *post hoc* attributions of own eating behavior (22) because participants’ perceived relevance aligns with empirical findings showing links between negative, but not positive, emotions and lapses in daily life [i.e., see meta-analysis in (8)]. Accordingly, positive and negative affect should not be treated as exerting opposing effects on dietary goal pursuit, but rather as distinct constructs with primarily negative affect playing a role in lapse occurrence.

Lastly, mapping fine-grained self-regulation constructs to prior EMA research was challenging. Prior classification based on the Theoretical Domains Framework for behavioral change (23) included self-regulation-related constructs such as behavioral regulation, beliefs about consequences and capabilities as well as memory and decision processes. These categories did not align clearly with the data-driven category structure identified here, raising the risk of jingle-jangle fallacies [i.e., constructs labeled differently or distinct constructs sharing labels (24)]. For instance, momentary intentions have been grouped under “memory and decision processes” but may correspond to “deliberate exceptions” in current analyses (e.g., when dietary intentions are temporarily suspended). Accordingly, we refrain from further interpretation, as such discrepancies in labeling could otherwise suggest that certain constructs (e.g., deliberate exceptions) have been neglected, when in fact they have been studied under different terminology.

### 4.2 Do reasons for dietary goal pursuit generalize across participants?

Next to qualitative identification of relevant factors, the repeated-measure design allowed systematic investigation of which reasons are consistently endorsed across participants, and which are more idiographic. Overall, findings indicated limited generalizability: no lapse category was reported at least once by more than 50% of participants (i.e., highest User Coverage was 47.73%), and for goal adherence, only two mid-level categories were endorsed by more than 80% of participants, with all remaining categories showing substantially lower coverage. Interpretation of this descriptive measure, however, depends critically on the underlying data basis, as higher reporting intensity increases the likelihood that a given category is endorsed at least once. Additional sensitivity analyses (see Supplement F) indeed showed that two mid-level lapse categories (social and physiological factors) also reached coverage levels of approximately 80% among users who contributed a relatively large number of lapse reports. As dietary lapses were generally reported less frequently, apparent differences in category endorsement between lapses and adherence thus likely reflect differences in reporting intensity rather than substantive differences in underlying processes. Further, while most EMA studies in the dietary domain employ similar sampling durations to the present study [i.e., between 7 and 14 days (25)], the pattern that User Coverage increases with underlying data basis (at least to a certain point), suggests that the manuscripts conclusions regarding limited generalizability primarily apply to study designs and populations with comparable data density. However, even within subsamples of participants with most reported reasons (see Supplement F), no category was endorsed by all participants, and especially more fine-grained (lower-level) categories were still typically endorsed by only a relatively small proportion of individuals (i.e., less than 50%).

This pattern aligns with prior research highlighting that predictors of eating episodes are highly idiographic. For example, in models predicting lapses, generalization of the group algorithm to each participant is usually poor [e.g., see (10)] and in the context of binge eating, highly individualized subsets of EMA items have been selected to achieve accurate person-level predictions (13). Further, using machine learning, an EMA study has identified six distinct subgroups of individuals characterized by different risk patterns for unhealthy eating [e.g., some participants were prone to unhealthy eating when being at home in the evening, and others by craving states regardless of context (26)]. Thus, present findings, together with prior work, suggest that fully standardized (nomothetic) item sets probably include multiple items irrelevant to parts of the sample. Incorporating personalized items (e.g., self-selected from a larger pool) may therefore improve the trade-off between participant burden and predictive utility of assessed factors.

However, differences in User Prevalence, Variance, and Top-User Probability between categories suggest that some factors would probably perform better within a nomothetic item set than others. Note that ‘nomothetic’ and ‘idiographic’ candidates here denote the most nomothetic- or idiographic-leaning categories within the extracted category system and the present sample, rather than properties expected to generalize across different coding schemes or samples. For lapses, cravings, social factors, and physiological factors were most consistently endorsed across participants; for adherence, lack of opportunity to lapse was. These factors, and their most frequently reported finer-grained categories (hunger, and being occupied), may thus be most suitable in a core nomothetic item set, which could be further enriched by more individual-specific items. Factors that appeared highly relevant across lapse occurrences in some, but not all, individuals included emotional factors, particularly stress (i.e., based on high User Variance and Top-User Probability). This aligns with evidence of inter-individual differences in emotion-, or stress-driven eating, whereby some individuals report decreased intake, others increased intake, and others no change (27, 28). Self-regulation-related factors also warrant particular attention in the context of dietary adherence. This category showed second highest Average User Prevalence and the highest Top-User Probability, indicating that it was highly relevant across and within individuals. However, its intermediate User Variance suggests a mixed pattern that does not clearly align with either a fully nomothetic or fully idiographic structure.

Analyzing User Prevalence, Variance, and Top-User Probability also informs intervention design. Dietary interventions targeting nomothetic factors are probably most effective at the population level, whereas idiographic factors may be better suited for tailored or adaptive components based on individual risk profiles. Moreover, when designing interventions for populations with limited self-regulatory capacities, researchers may specifically focus on indices of subcategories of external factors to identify the most relevant intervention targets to support dietary goal adherence. Conversely, when environmental modification is not feasible, a more detailed examination of self-regulation subcategories may be possible. For example, self-control could be a particularly promising target, given its highest Average User Prevalence and lowest User Variance within this mid-level category. We did not additionally analyze active (i.e., self-regulation-related) and passive (i.e., opportunity-related) adherence reasons as separate outcomes, as the inductively derived mid-level category structure already keeps these processes distinct and all indices are reported separately per category.

### 4.3 Strength and limitations

A strength of this mixed-methods study is that reasons were assessed for both lapses and adherence. While related factors are typically assumed to operate in opposite directions (i.e., absence of lapse risk factors should increase adherence, and vice versa), assessing both likely elicited distinct though processes and captured complementary perspectives. In line with this, some factors (e.g., hunger, desire levels, availability of goal-(in)congruent foods) were reported for both, whereas others were specific to either adherence or lapses (e.g., routines, specific strategies, or unplanned circumstances). Another strength is that qualitative responses were collected immediately after lapse and goal adherence in daily life. This should reduce retrospective recall biases and increase sensitivity to context-dependent determinants which may be overlooked in memory-based accounts. Indeed, in contrast to previous qualitative work based on focus groups (5), the current analyses highlighted a broader range of external factors.

A limitation is that reports were likely constrained by response burden. Although participants were instructed to report all relevant reasons, they often provided only a single reason per entry and interrater reliability for the number of reasons coded per entry was only moderate. Consequently, less salient but still relevant factors may be underreported and, when present in open-text responses, inconsistently identified as distinct reasons across coders. This may have resulted in downward bias in estimates of rarer categories and potentially attenuated their observed generalizability across individuals.

Another limitation is that this study employed ‘Conversational Analysis to the power of AI’ (CA^AI^) an AI-supported approach to qualitative content analysis, which is well suited for the theory-guided and systematic handling of large textual datasets (16). However, its category-based reduction may lead to a loss of nuance, potentially limiting the richness of individual cases and perspectives (17). Moreover, qualitative content analysis approaches whether AI-supported or traditional are limited in their ability to capture deeper, latent structures within texts (17). However, no responses were assigned to a “rest” category, suggesting that the established coding scheme was nuanced enough to sufficiently captured all reported reasons.

Relatedly, AI-generated thematic structures may be biased, for instance toward more frequently expressed content or by the model itself. We sought to mitigate this in two ways: in QInsights, the Retrieval-Augmented Generation architecture restricted theme identification to the uploaded responses rather than the model’s training data, and all AI-generated themes and categories were verified and, where necessary, revised through researcher oversight (two rounds of communicative validation and independent double-coding with intercoder reliability). Moreover, it should be kept in mind that we had no pre-defined cut-offs to select categories most suitable as nomothetic or idiographic predictors, but selected categories with highest Average User Prevalence and lowest User Variance as potential nomothetic predictors and categories with highest User Variance and highest Top-User Probability as potential idiographic predictors. Thus, selection of the most suitable categories was based on relative ranking across observed patterns and is thus sensitive to the specific context (e.g., number of meaningful categories or average level of indices across categories). Related to this, the qualitative analyses were not pre-registered. Potential *post hoc* analysis biases [HARKing, see Kerr, (30)] can, therefore, not be ruled out.

Regarding generalizability, it should be noted that the sample consisted predominantly of females recruited via social media posts and university course announcements in German-speaking countries. As such, the findings may not be fully generalizable to more gender-balanced or predominantly male dieting populations and to broader cultural and socio-economic contexts. Furthermore, due to instructions, most reported goals involved intake restriction, and therefore findings may not extend to goals involving increased intake, such as increasing protein or vegetable consumption.

## 5 Conclusion

Dieters’ self-reported reasons extended beyond commonly used EMA predictors. In addition to typically assessed factors, they highlighted health-related states, the availability of goal-*con*gruent alternatives, specific opportunity-related factors as well as a more balanced role of internal and external factors in general. Cravings, social influences, physiological states (especially hunger), and lack of opportunity to lapse (especially being occupied) appeared most broadly generalizable across participants and may be most suitable as nomothetic predictors for dietary goal pursuit in future research. However, substantial idiographic variability (e.g., for emotion-related factors) suggests that fully standardized EMA item pools may be insufficient and justify future studies testing if individualized item selection can improve prediction of dietary lapses and just-in-time adaptive interventions.

## Data Availability

Publicly available datasets were analyzed in this study. The data for the current analyses stem from two EMA studies and can be found on OSF: https://osf.io/fapwc.

## Footnotes

1
https://osf.io/fapwc

2 The working language was German; the following are translations of the prompts and categories.

3 On 13 entries (i.e., 0.2%) stress was selected as a category as an adherence reason, even though this category was only present for lapses according to the coding system. These were recoded as in “occupied” in “lack of opportunity” which specifically gave “time stress” as an example and reported reasons included for example “time stress,” “learning stress” or “stressed about an appointment, so I did not eat anything.”
