# Supplementary material for: Nomothetic and idiographic determinants of dietary lapses and adherence: an exploratory mixed methods ecological momentary assessment study of open-text responses
Source: Front Nutr. 2026 Aug 14;13:1869531. doi: 10.3389/fnut.2026.1869531 (PMC13521853; doi:10.3389/fnut.2026.1869531)
Supplement: Supplementary file 1 [file Supplementary_file_1.zip › Supplement A.docx]

**Supplement A: List of All Self-reported Dietary Goals**

I want to avoid sweets; I want to reduce my body weight to 60 kg by reducing my consumption of sweets and alcohol; I want to eat fewer unhealthy snacks; I want to consume no more than 1700 kcal per day; I want to eat less bread and more vegetables and legumes; I want to consume no more than 1800 kcal per day; I want to avoid unhealthy snacks (products with added sugar or chips etc.); I want to eat regular meals and never overeat, reduce industrial sugar intake significantly, and avoid sugar 3 hours before sleep; I want to reduce my calories by about 200 kcal below my daily requirement; I want to eat three proper meals per day and snack less; I want to consume more calories to reach a daily requirement of 2200 kcal in order to support my goal of building lean muscle mass; I want to eat no sweets; I want to eat less sugar; I want to eat fewer sweets; I want to eat a higher-protein diet; I want to consume more than 30g of protein per day; I want to avoid sweets during the day and reduce carbohydrates (grains, dairy products); I want to consume a maximum of 1498 kcal per day; I want to consume no more than 1650 kcal per day; I want a target of 2400 kcal; I want a target of 1500 kcal; I want to avoid sweets and Kaba (cocoa drink) until Easter; I want to eat more fruit and vegetables and have a healthier diet; I want to avoid sugar, especially in sweets and sugary drinks; I want to eat fewer cookies and sweets during working hours; I want to eat sweets at most once every two days; I want to eat less sugar and fat; I want to avoid sweets; I want to avoid sweets until Easter; I want to consume soft drinks only with one meal per day; I want to consume no more than 2000 kcal per day; I want to reduce snacking between meals to twice per week and control portion sizes; I want to completely avoid sweets; I want to consume no more than 1300 kcal per day; I want to avoid unnecessary sugar; I want to avoid soft drinks and limit sweets to a small portion per day; I want to eat no sweets; I want to reduce sugar and replace snacks with healthier options; I want to eat less than 1900 kcal; I want to reduce coffee and sweets consumption; I want to reduce my sugar intake; I want to reduce calories (below 1800 kcal) and limit unhealthy foods (chips, ready meals) to once per week, and only eat raw vegetables as snacks; I want to avoid sweets and eat no more than 1300 kcal per day; I want to reduce dairy consumption; I want to eat little wheat, almost none; I want to avoid energy drinks and stop afternoon coffee and eat only one hot meal per day; I want to eat meat no more than twice per week; I want to stop eating snacks between meals and avoid unnecessary eating; I want to eat healthier; I want a minimum of 1500 kcal per day, a 200–500 kcal deficit, over 100g protein daily, max 2 snacks per day, and fruit/vegetables daily; I want to snack less and reduce sugar; I want to eat unprocessed foods and lose 6 kg; I want no sweets; I want to eat less meat; I want to avoid sweets during this period; I want to reduce alcohol and sweets and consume a maximum of 2000 kcal per day with a high-protein balanced diet; I want to eat no meat; I want to gain about 10 kg due to being underweight, have a strong lactose intolerance and avoid foods I cannot tolerate, and replace cow’s milk with plant-based milk while struggling with stress-related intuitive eating; I want to eat more food (around 2500–3000 kcal), preferably nutrient-dense; I want to eat more consciously and avoid sweets; I want to eat fewer sweets; I want to eat more protein; I want to lose 5 kg and exercise more; I want to lose weight; my plan is a 4-week broth diet with only vegetable broth, water, and occasional apples; I want regular meals and more nutrients; I want to reduce sweets and fat intake; I want to avoid gut-unfriendly foods; I want to eat fewer gummy bears and sweets; I want to avoid animal products; I want to avoid heavily processed foods such as instant noodles, frozen pizza, chips, and sweets; I want to lose 3 kg; I want to drink less soda (Spezi) and not eat after 11 pm; I want to eat fewer sweets; I want to avoid sugar and inflammatory foods due to neurodermatitis; I want to stop eating after dinner; I want to eat less sweets; I want to reduce sugar and reduce my weight; I want to consume no more than 1500 kcal per day; I want to eat less fat and sugar; I want to eat in a calorie deficit with less sugar; I want to snack less, avoid overeating, and recognize satiety earlier; I want fewer sweets; I want no sweets (including cake); I want fewer carbohydrates and a maximum of 1800 kcal; I want to eat fewer calories; I want to reduce reliance on ready-made foods; I want to eat less bread and more fruit and vegetables; I want to eat fewer unhealthy snacks and sweets; I want fewer sweets; I want no snacks between meals except fruit and vegetables; I want no more than 1300 kcal per day; I want to eat at least 3 meals per day; I want not to eat after dinner; I want to avoid sugar and wheat flour; I want to avoid sweets and unhealthy snacks like chips; I want to avoid snacks between meals; I want to eat no more than 1400 kcal; I want no sweets; I want to avoid sweets; I want to reduce meat consumption; I want to consume less than 1700 kcal; I want to minimize carbohydrate intake; I want a calorie deficit; I want to eat fruit daily; I want to eat healthier; I want to eat vegetables every day; I want a balanced diet and no sweets; I want to consume no more than 1100 kcal per day; I want to eat fewer sweets and avoid snacks; I want to lose weight and eat no more than 1800 kcal; I want not to snack between meals; I want to eat fewer than 2000 kcal per day; I want fewer calories and fewer binge eating episodes; I want to practice 16:8 intermittent fasting; I want to reduce sugar and try intermittent fasting (16:8); I want to eat more regularly but less overall; I want to consume no more than 1400 kcal per day; I want a more balanced and protein-rich diet; I want to eat only when hungry and avoid sweets and snacking; I want to eat slightly less but protein-rich; I want fewer sweets; I want a maximum of one sweet per day; I want to consume no more than 1500 kcal per day; I want no alcohol and fewer sweets; I want to eat more in the morning; I want to reduce processed foods; I want to reduce sugar and sugar substitutes; I want to reduce snacking and processed foods; I want to eat no sugar; I want to consume no more than 1600 kcal; I want less sugar; I want to avoid ready meals; I want no sugar; I want to eat 20% less; I want fewer sweets; I want to eat as little industrial sugar as possible; I want no chips and chocolate; I want to avoid sweets and reduce sugar intake; I want to reduce binge eating to maintain a calorie deficit and lose weight; I want to eat a healthy and balanced diet; I want to eat fewer sweets and less meat; I want fewer sweets; I want a maximum of 100g sweets per week; I want no more than 2000 kcal per day; I want no sweets or sugary drinks for a week; I want no soft drinks; I want no more than 1500 kcal per day and a smoothie instead of dinner; I want a balanced diet and no sweets or chips; I want to reduce sugar in general; I want no sweets and less fatty foods; I want to reduce sweets consumption; I eat everything and try to stay below 2500 kcal; I want to reduce binge eating, improve my relationship with food, gain weight gradually, and improve mental health and exercise habits with structured meals and exposure to previously “forbidden” foods; I want to eat less sugar and not more than 2000 kcal; I want to prepare breakfast at home instead of buying it and only eat sweets on Saturdays; I want to eat fewer unhealthy foods and eat more intuitively; I want to reduce sugar intake and avoid soft drinks more often; I want to avoid ultra-processed sweets and baked goods; I want no more than 1900 kcal; I want to eat less sweet and fatty foods; I want to lose 1 kg with a daily 500 kcal deficit; I want to avoid sugar; I want to eat healthier; I want to reduce unhealthy sugar; I want to eat smaller portions to lose weight; I want to eat more regional and healthy foods; I do not want to eat when I am not hungry; I want no more than 1500 kcal per day (adjusted for activity level); I want no more than 2000 kcal per day and daily fruit/vegetables; I want to reduce sugar and increase protein while improving dietary balance and reducing on-the-go eating; I want to meet my daily protein needs and eat a healthy diet.
