# Supplementary material for: Nomothetic and idiographic determinants of dietary lapses and adherence: an exploratory mixed methods ecological momentary assessment study of open-text responses
Source: Front Nutr. 2026 Aug 14;13:1869531. doi: 10.3389/fnut.2026.1869531 (PMC13521853; doi:10.3389/fnut.2026.1869531)
Supplement: Supplementary file 1 [file Supplementary_file_1.zip › Supplement B.docx]

**Supplement B: Additionally Assessed Questionnaires**

After reporting their dietary goal, participants rated the importance and motivation to pursue their goal (from 0 = "not at all" to 100 = "very"), as well as the perceived difficulty of goal attainment (from 0 = "very easy" to 100 = "very difficult"). They then completed the following questionnaires: the Perceived Self-regulatory Success in Dieting Scale (PSRS; Meule et al., 2012), the Eating Disorder Examination-Questionnaire (EDE-Q; Kliem et al., 2016), the Inflexible Eating Questionnaire (IEQ; Schultz et al., 2022), and the short version of the Self Compassion Scale (Hupfeld & Ruffieux, 2011; Raes et al., 2011) in both Studies, as well as an adapted version of the Stress Mindset Measure (Crum et al., 2013) in Study 1 only, and the Emotion Regulation Questionnaire (Abler & Kessler, 2009) in Study 2 only.
