# Supplementary material for: Nomothetic and idiographic determinants of dietary lapses and adherence: an exploratory mixed methods ecological momentary assessment study of open-text responses
Source: Front Nutr. 2026 Aug 14;13:1869531. doi: 10.3389/fnut.2026.1869531 (PMC13521853; doi:10.3389/fnut.2026.1869531)
Supplement: Supplementary file 1 [file Supplementary_file_1.zip › Supplement C.docx]

**Supplement C: Five Lists and Coding Manual**

**C1. Five Theme Lists**

Welche Themen werden in den Gründen für die Nicht-Einhaltung oder die Einhaltung von Ernährungszielen genannt? Trenne die Themen nach Einhaltung und Nicht-Einhaltung. Führe jeweils 3-8 Themen für Einhaltung und Nicht-Einhaltung auf, je nach dem, wie viele du jeweils findest. Es müssen nicht gleich viele Themen für Einhaltung und Nicht-Einhaltung der Ernährungsziele sein. Erstelle für jedes gefundene Thema einen kurzen Erklärtext. Füge für jedes gefundene Thema zwei Beispiele aus den Daten an. Antworte auf deutsch.

**Answers 2-1001**

**Einhaltung der Ernährungsziele**

- **Kein Verlangen:** Viele Teilnehmer geben an, dass sie kein Verlangen nach ungesunden Lebensmitteln hatten, was ihnen half, ihre Ziele einzuhalten. Beispiele: "Kein Verlangen" (File: Gründe 2-1001.xlsx), "Kein Verlangen nach Zucker" (File: Gründe 2-1001.xlsx).
- **Ablenkung:** Ablenkung durch Arbeit, Uni oder andere Aktivitäten wird häufig als Grund für die Einhaltung genannt. Beispiele: "Ich war in der Uni" (File: Gründe 2-1001.xlsx), "Ich war abgelenkt durch die Arbeit" (File: Gründe 2-1001.xlsx).
- **Gesunde Alternativen:** Einige Teilnehmer haben gesunde Alternativen gewählt, um ihre Ziele zu erreichen. Beispiele: "Gesundes Abendessen gekocht" (File: Gründe 2-1001.xlsx), "Ich habe meinen Hunger mit nahrhaften gesunden Lebensmitteln gestillt" (File: Gründe 2-1001.xlsx).
- **Selbstdisziplin:** Selbstdisziplin und Motivation werden oft als Gründe für die Einhaltung genannt. Beispiele: "Selbstdisziplin" (File: Gründe 2-1001.xlsx), "Ich habe mir fest vorgenommen das Ziel einzuhalten" (File: Gründe 2-1001.xlsx).
- **Keine Verfügbarkeit von ungesunden Lebensmitteln:** Wenn ungesunde Lebensmittel nicht verfügbar sind, fällt es den Teilnehmern leichter, ihre Ziele einzuhalten. Beispiele: "Nichts da" (File: Gründe 2-1001.xlsx), "war nichts da" (File: Gründe 2-1001.xlsx).

***Aus Runde 1 (ohne Erklärtext):***

- **Gesundheitliche Gründe** (z.B. "Ich habe geschlafen", "Mir ging es gesundheitlich nicht gut").
- **Regelmäßige Mahlzeiten und Sättigung** (z.B. "Gut gefrühstückt", "Durch reguläre Mahlzeiten satt").

**Nicht-Einhaltung der Ernährungsziele**

- **Stress:** Stress wird häufig als Grund für die Nicht-Einhaltung genannt. Beispiele: "Stress" (File: Gründe 2-1001.xlsx), "Stress, lange Zeit zuvor nichts gegessen" (File: Gründe 2-1001.xlsx).
- **Aus Runde 1:** Fehlende Zeit oder Gelegenheit (z.B. "Keine Zeit zu kochen", "Nachtschicht").
- **Verfügbarkeit von ungesunden Lebensmitteln:** Die Verfügbarkeit von ungesunden Lebensmitteln führt oft zu Rückfällen. Beispiele: "Verf fcgbarkeit" (File: Gründe 2-1001.xlsx), "S fc digkeiten wurden ausgeteilt" (File: Gründe 2-1001.xlsx).
- **Emotionale Gründe:** Emotionale Zustände wie schlechte Laune oder Frust führen häufig zu Rückfällen. Beispiele: "Schlechte Stimmung, M fcdigkeit" (File: Gründe 2-1001.xlsx), "Starke Emotionen, gedr fcckte Stimmung" (File: Gründe 2-1001.xlsx).

**Aus Runde 1:** Feiern und soziale Anlässe

- **Gesellschaftlicher Druck:** Gesellschaftlicher Druck oder Gruppenzwang wird ebenfalls als Grund für die Nicht-Einhaltung genannt. Beispiele: "Lust und Gruppenzwang" (File: Gründe 2-1001.xlsx), "Mitessen in Anwesenheit anderer" (File: Gründe 2-1001.xlsx).
- **Hei dfhunger:** Hei dfhungerattacken führen oft dazu, dass Teilnehmer ihre Ziele nicht einhalten. Beispiele: "Hei dfhunger" (File: Gründe 2-1001.xlsx), "konnte dem Hei dfhunger nicht mehr widerstehen" (File: Gründe 2-1001.xlsx).

**Answers 1002-2001**

**Einhaltung der Ernährungsziele**

- **Motivation:** Die innere Motivation hilft den Teilnehmern, ihre Ziele zu erreichen. Beispiele: "Motivation, gut geschlafen" und "Motivation, Abendessen".
- **Verfügbarkeit von Alternativen:** Teilnehmer greifen auf gesunde Alternativen zurück, um ihre Ziele zu erreichen. Beispiele: "gute Alternativen verfügbar" und "Alternativen zur Verfügung".
- **Disziplin:** Selbstkontrolle und Disziplin sind entscheidend für die Zielerreichung. Beispiele: "Disziplin" und "Disziplin, beschäftigt mit Uni".
- **Planung und Vorbereitung:** Teilnehmer bereiten ihre Mahlzeiten vor, um ihre Ziele zu erreichen. Beispiele: "Essen bereits vorbereitet" und "habe mir einen Plan gemacht und entsprechend gekocht". Aus Runde 1: **Regelmäßige Mahlzeiten**
- **Kein Verlangen nach ungesunden Lebensmitteln:** Das Fehlen von Appetit auf ungesunde Lebensmittel unterstützt die Zielerreichung. Beispiele: "kein Verlangen danach" und "kein Bedürfnis".

**Nicht-Einhaltung der Ernährungsziele**

- **Stress:** Stress führt dazu, dass Teilnehmer ihre Ziele nicht einhalten können. Beispiele: "Stress und dadurch zu Lebensmitteln gegriffen, die schnell zugänglich sind" und "viel gelernt, keine Zeit".
- **Hunger und Heißhunger:** Starker Hunger oder Heißhunger erschwert die Einhaltung der Ziele. Beispiele: "Sehr hungrig" und "Heißehunger Attacke".
- **Gesellschaft und soziale Anlässe:** Soziale Situationen wie Feiern oder Treffen mit Freunden führen zu Rückfällen. Beispiele: "Gesellschaft" und "Treffen mit Freunden".
- **Fehlende Alternativen:** Das Fehlen gesunder Alternativen erschwert die Zielerreichung. Beispiele: "keine Alternativen zur Verfügung und ich war unterwegs" und "spontanes Essen gehen, keine Alternativen".
- **Emotionale Gründe:** Emotionen wie Frust oder Traurigkeit führen zu Rückfällen. Beispiele: "Frust" und "Traurig".

**Aus Runde 1:**

- **Müdigkeit und Unwohlsein:** Beispiele: "Müdigkeit, Unwohlsein" (Gründe 1002-2001.xlsx), "bin eingeschlafen und habe nichts gegessen" (Gründe 1002-2001.xlsx).
- **Verlockung durch Essen:** Beispiele: "Mamas Kuchen war einfach zu gut" (Gründe 1002-2001.xlsx), "wenn ich meine Tage krieg, hab ich immer Gelüste nach Milchprodukten" (Gründe 1002-2001.xlsx).
- **Fehlende Zeit:** Beispiele: "viel gelernt, keine Zeit" (Gründe 1002-2001.xlsx), "hatte Uni und hab nichts gegessen" (Gründe 1002-2001.xlsx).

**Answers 2002-3001**

**Einhaltung der Ernährungsziele**

**Motivation:** Viele Teilnehmer nannten Motivation als Grund, ihre Ziele einzuhalten. Dies könnte durch externe Faktoren wie Freunde oder durch interne Faktoren wie den Wunsch nach Erfolg ausgelöst werden. Beispiel: "Meine Freundin hat mich motiviert." Beispiel: "Motivation durch diese Studie um mir selbst etwas zu beweisen."

**Disziplin:** Disziplin wurde oft als Schlüssel zur Einhaltung genannt. Teilnehmer berichteten, dass sie sich bewusst an ihre Ziele hielten. Beispiel: "Diszipliniert, und nicht zur Verfügung." Beispiel: "Disziplin und keine Gelegenheit."

**Planung:** Eine gute Planung half den Teilnehmern, ihre Ziele zu erreichen, indem sie Mahlzeiten vorbereiteten oder gesunde Alternativen wählten. Beispiel: "Essen vorbereitet." Beispiel: "Planung, Disziplin."

**Keine Verfügbarkeit von ungesunden Lebensmitteln:** Teilnehmer berichteten, dass das Fehlen von Versuchungen ihnen half, ihre Ziele einzuhalten.

Beispiel: "Keine Süßigkeiten greifbar." Beispiel: "Keine Schokolade im Kühlschrank."

**Gesunde Alternativen:** Der Konsum von gesunden Alternativen wie Obst oder Gemüse wurde als hilfreicher Faktor genannt.

Beispiel: "Obst als Ersatz für Süßigkeiten." Beispiel: "Gesundes Frühstück mit Obst und Protein steht am Plan."

**Nicht-Einhaltung der Ernährungsziele**

**Verfügbarkeit von Versuchungen:** Die Verfügbarkeit von ungesunden Lebensmitteln oder Snacks führte oft zu Rückfällen.

Beispiel: "Essen war so verlockend." Beispiel: "Schokolade war noch offen."

**Emotionale Faktoren:** Emotionale Zustände wie Stress, Frustration oder schlechte Laune wurden häufig als Gründe für Rückfälle genannt. Beispiel: "Stress wegen Termin, deshalb nichts gegessen." Beispiel: "Schlechte Laune."

**Gesellschaftlicher Druck:** Soziale Situationen wie Geburtstagsfeiern oder Einladungen führten oft zur Nicht-Einhaltung. Beispiel: "Geburtstagsfeier von einer Freundin." Beispiel: "Gesellschaftlicher Druck, wäre unhöflich gewesen."

**Schwache Impulskontrolle:** Teilnehmer berichteten, dass sie ihren Impulsen nicht widerstehen konnten. Beispiel: "Schwache Impulskkontrollr heute." Beispiel: "Hatte Lust auf Essen und meine Freundin hat gekocht."

**Fehlende Alternativen:** Das Fehlen gesunder Alternativen wurde ebenfalls als Grund für Rückfälle genannt. Beispiel: "Keine Alternative vorhanden." Beispiel: "Schlechte Alternativen vorhanden."

**Answers 3002-4001**

**Einhaltung der Ernährungsziele**

**Willenskraft:** Viele Teilnehmer nannten ihre Willenskraft als entscheidenden Faktor, um ihre Ziele zu erreichen. Beispiele: "Willenskraft!!!" (Gründe 3002-4001.xlsx), "Willenskraft" (Gründe 3002-4001.xlsx).

**Gesunde Alternativen:** Die Verfügbarkeit gesunder Alternativen half den Teilnehmern, ihre Ziele einzuhalten. Beispiele: "Gesunde Alternativen" (Gründe 3002-4001.xlsx), "vegetarische alternative" (Gründe 3002-4001.xlsx).

**Motivation:** Die innere Motivation und das Ziel vor Augen zu haben, wurden oft als Gründe für die Einhaltung genannt. Beispiele: "Motivation" (Gründe 3002-4001.xlsx), "Bin heute besonders motiviert" (Gründe 3002-4001.xlsx).

**Beschäftigung/Ablenkung:** Ablenkung durch Arbeit oder andere Aktivitäten wurde als hilfreicher Faktor genannt. Beispiele: "Arbeit" (Gründe 3002-4001.xlsx), "Ablenkung" (Gründe 3002-4001.xlsx).

**Routine:** Eine etablierte Routine, wie ein gesundes Frühstück, unterstützte die Einhaltung. Beispiele: "Routine (gesundes Frühstück)" (Gründe 3002-4001.xlsx), "Routine" (Gründe 3002-4001.xlsx).

**Nicht-Einhaltung der Ernährungsziele**

**Sozialer Druck:** Essen in Gesellschaft oder soziale Verpflichtungen führten oft zu Rückfällen. Beispiele: "Geburtstagsfeier" (Gründe 3002-4001.xlsx), "Mit Freunden zusammen" (Gründe 3002-4001.xlsx).

**Stress:** Stress und Frustration wurden häufig als Gründe für die Nicht-Einhaltung genannt. Beispiele: "Stress" (Gründe 3002-4001.xlsx), "Frust" (Gründe 3002-4001.xlsx).

**Verfügbarkeit ungesunder Lebensmittel:** Die einfache Verfügbarkeit von ungesunden Lebensmitteln führte zu Rückfällen. Beispiele: "Nahrungsmittel war in der Nähe" (Gründe 3002-4001.xlsx), "Süßigkeiten im Büro" (Gründe 3002-4001.xlsx).

**Selbstsabotage:** Einige Teilnehmer nannten Selbstsabotage als Grund für Rückfälle. Beispiele:

**Müdigkeit:** Müdigkeit und Erschöpfung führten dazu, dass Teilnehmer ihre Ziele nicht einhalten konnten. Beispiele: "Müdigkeit" (Gründe 3002-4001.xlsx), "Müdigkeit" (Gründe 3002-4001.xlsx).

**Answers 4002-4900**

**Einhaltung der Ernährungsziele**

- **Disziplin und Motivation:** Viele Teilnehmer gaben an, dass ihre Disziplin und Motivation ihnen halfen, ihre Ernährungsziele einzuhalten. Beispiele: "Ich will mein Ziel erreichen" (UserId: NKvL-hmS4-ciKJ) und "Motivation, keine Lust und Zeit" (UserId: 5sRR-zASi-kbyA).
- **Planung und Routine:** Eine gute Planung und das Einhalten von Routinen wurden oft als Gründe für die Einhaltung genannt. Beispiele: "Routine, klares Ziel" (UserId: cTMw-RBHp-2aw9) und "bessere Planung" (UserId: REAh-RUdQ-L3Af).
- **Kein Hunger oder kein Verlangen:** Einige Teilnehmer hielten ihre Ziele ein, weil sie keinen Hunger oder kein Verlangen nach bestimmten Lebensmitteln hatten. Beispiele: "Kein Hunger" (UserId: rYmf-Lf84-mXj9) und "Kein Verlangen" (UserId: V53f-BsHq-hbku).
- **Gesunde Alternativen:** Die Verfügbarkeit von gesunden Alternativen half den Teilnehmern, ihre Ziele zu erreichen. Beispiele: "Gesunde Alternativen zuhause" (UserId: 3xyz-G11r-CX3q) und "Leckere Alternative" (UserId: rYmf-Lf84-mXj9).
- **Unterstützung durch andere:** Unterstützung durch Familie oder Freunde wurde ebenfalls als Grund genannt. Beispiele: "Meine Familie hat mich unterstützt" (UserId: 3XFS-hJDb-Te38) und "Selbst mit Freundin gekocht, die auch ungern Fleisch zubereitet" (UserId: bVED-wYJ3-wV6q).

**Nicht-Einhaltung der Ernährungsziele**

- **Stress:** Stress war ein häufiger Grund für die Nicht-Einhaltung der Ziele. Beispiele: "Stress" (UserId: sjkD-gp8P-uME6) und "Stress, Anspannung" (UserId: TWjA-WCn1-MEYF).
- **Hunger und Appetit:** Viele Teilnehmer gaben an, dass Hunger oder Appetit sie dazu brachten, ihre Ziele nicht einzuhalten. Beispiele: "Hunger" (UserId: NKvL-hmS4-ciKJ) und "Appetit auf Süßes" (UserId: rQef-jspr-W6x1).
- **Verfügbarkeit von ungesunden Lebensmitteln:** Die Verfügbarkeit von ungesunden Lebensmitteln führte oft zu Rückfällen. Beispiele: "Süßigkeiten zuhause gehabt" (UserId: 3xyz-G11r-CX3q) und "Es gab Donuts bei der Arbeit" (UserId: zV6G-B3dD-Ghfn).
- **Soziale Einflüsse:** Soziale Situationen wie Treffen mit Freunden oder Familienessen wurden als Hindernisse genannt. Beispiele: "Familienessen" (UserId: 8K36-ahRB-aJaw) und "Freunde" (UserId: zfYq-cFFv-zvSv).
- **Emotionale Gründe:** Emotionen wie Frust oder Langeweile führten ebenfalls zu Rückfällen. Beispiele: "Frust" (UserId: rYmf-Lf84-mXj9) und "Langeweile" (UserId: H3er-jmrA-r7kL).

| **Einhaltung von Ernährungszielen** | **Dietary lapses** |
| --- | --- |
| **INTERN** | **INTERN** |
| **Selbstregulation** | **Selbstregulation** |
| Selbstkontrolle | Fehlende Selbstkontrolle |
| Motivation | Fehlende Motivation |
| Planung und Vorbereitung | Fehlende Planung |
| Routinen | Geplante / bewusste Ausnahme |
| Spezifische Strategien |  |
|  | |
| **Emotionale Faktoren** | **Emotionale Faktoren** |
|  | Unangenehme Gefühle und Langweile |
|  | Unterstützung zur Zustandsverbesserung |
|  | Stress |
|  | |
| **Physiologische Faktoren** | **Physiologische Faktoren** |
| Gesundheitszustand | Gesundheitszustand |
| Schlafbezogene Faktoren | Schlafbezogene Faktoren / Erschöpfung |
| Hunger | Hunger |
|  | |
| **Verlangen** | **Verlangen** |
|  | |
| **EXTERN** | **EXTERN** |
| **Fehlende Gelegenheiten** | **Zeitmangel und Gelegenheiten** |
| Beschäftigt |  |
| Ortsbedingt / Unterwegs |  |
| Geschlafen |  |
| Andere Faktoren |  |
|  | |
|  | **Ungeplante Umstände** |
|  | |
| **Verfügbarkeiten von Lebensmitteln** | **Verfügbarkeiten von Lebensmitteln** |
| Fehlende Verfügbarkeit zu vermeidender Lebensmittel | Einfache Verfügbarkeit zu vermeidender Lebensmittel |
| Einfache Verfügbarkeit von Alternativen | Fehlende Verfügbarkeit von Alternativen |
|  | |
| **Soziale Faktoren und externe Motivation** | **Soziale und gesellschaftliche Faktoren** |
|  | |
| **ANDERES** | **ANDERES** |
| **Sonstiges** | **Sonstiges** |
|  | |
| **Keine Angabe von Gründen** | **Keine Angabe von Gründen** |

**C2. Coding Manual**

Bitte das Kategorienhandbuch vor dem Codieren sorgfältig durchlesen

- Ein Aspekt in einem Textsegment wird eindeutig einer Kategorie zugewiesen (Ausnahme Stress, s. Handbuch), Mehrfachzuordnungen eines Aspekts sind nicht vorgesehen.
- Ausnahme: wenn in einem Textsegment mehrere *Aspekte* thematisiert sind, wird das Segment auch mehreren Kategorien zugewiesen, z.B. wird „Disziplin und keine Gelegenheit“ den Kategorien ‚Selbstregulation\Selbstkontrolle‘ sowie ‚Fehlende Gelegenheiten\Beschäftigt‘ zugewiesen.
- Sollte absolut keine Zuordnung möglich ist, wird der Aspekt / das Textsegment der Kategorie „Sonstiges“ zugeordnet. Diese Kategorie bitte sparsam und nur zur Not verwenden.
- Es wird diejenige Zuordnung gewählt, die am wenigsten der Interpretation der Forscherin / des Forschers bedarf.
- Wenn die Zuordnung zunächst uneindeutig erscheint, hilft es, sich das Ziel der befragten Person nochmal anschauen und sich einen ganzen Satz zu formulieren: „Ich habe mein Ernährungsziel, mehr Proteine zu essen, eingehalten, weil ich…..“. Dadurch wird die Zuordnung oftmals eindeutiger für die Forscherin / den Forscher.

**Kategorienhandbuch für die Einhaltung von Ernährungszielen**

INTERNE Gründe für die Einhaltung von Ernährungszielen haben unmittelbar mit dem eigenen Ernährungsverhalten einer Person zu tun. Dazu gehören:

1. Selbstregulation

*Diese Kategorie umfasst die inneren Beweggründe, die es Teilnehmenden ermöglichen ihre Ernährungsziele einzuhalten, sei es durch persönlichen Wunsch, Erfolgserlebnisse oder Selbstkontrolle. Auch Strategien zur (Umgehung von) Selbstregulation sind enthalten, wie Planung, Vorbereitung und spezifische Maßnahmen wie Kaorien-Tracking.*

1.1. Selbstkontrolle , z.B. „Selbstdisziplin“, „Diszipliniert,[ und nicht zur Verfügung]“, „Willenskraft!!!“, „Disziplin [und keine Gelegenheit]“
1.2. Motivation, auch durch Erfolgserlebnisse oder die eigene Zielorientierung,

z.B. „Bin heute besonders motiviert“, „Ich will mein Ziel erreichen“, „Zielstrebig“, „Habe mich an meine Ziele erinnert!“, „Motivation“, „Sommer kommt“, „Kleidung“

1.3. Planung und Vorbereitung, z.B. „Gesundes Abendessen gekocht“, „Essen bereits vorbereitet“, „Planung, [Disziplin]“, „Ernährungsplan“, „Mitgenommenes Mittagessen bei der Arbeit“, „Habe mit selbst Proteinreiche Jause mitgenommen“, „Ich hatte am Vortag schon ausreichend laktosefreies Essen zubereitet“
1.4. Routinen, z.B. „Routine (gesundes Frühstück)“, „normales Frühstück“, „normales Mittagessen“, „Mittlerweile entwickle ich wohl langsam eine Gewohnheit.“
1.5. Spezifische Strategien, z.B. „Tracker“, „Kalorien Zähler App“, „16-8 diät“, „achtsames Essen“, „Hunger mit etwas Nahrhaftem gestillt“, gezielte Ablenkung

2. Emotionale Faktoren

*Umfasst Emotionen, deren Abwesenheit sowie die Eigenschaften von Emotionen,*

z.B. „Keine Langeweile“, „zu faul zu kochen“, „kein Stress“, „schlechtes Gewissen“

3. Physiologische Faktoren

Diese Kategorie umfasst körperliche und gesundheitliche Faktoren, die das Essverhalten beeinflussen, wie den physischen Gesundheitszustand oder das Hunger- und Sättigungsgefühl.

3.1. Gesundheitszustand, z.B. „Schmerzfrei“, „Hangover“, „Krankheit“, „Periode“
3.2. Schlafbezogene Faktoren, z.B. "[Motivation,] gut geschlafen", „Müdigkeit“, „zu müde zum Essen“
3.3. Hunger, umfasst Aspekte von Hunger und Sättigung (je nach Ernährungsziel), z.B. "Gut gefrühstückt“, "Durch reguläre Mahlzeiten satt", "Kein Hunger", „Voll gegessen vom Mittag“

4. Verlangen

*Hier geht es um die Thematisierung von Verlangen, Lust und Appetit oder die Abwesenheit dieser. Es kann um bestimmte Lebensmittel oder Essen generell gehen.*

z.B. "Kein Bedürfnis", „keinen Gedanken daran gehabt“, „nicht daran gedacht“, „Verlangen nach gesunder Mahlzeit“, „keine Lust“, „Kein Interesse an Süßem“

EXTERNE Gründe für die Einhaltung von Ernährungszielen haben nicht unmittelbar mit dem eigenen Ernährungsverhalten einer Person zu tun, sondern sorgen von außen für die Einhaltung der Ziele. Dazu gehören:

5. Fehlende Gelegenheiten

*Hier geht es um fehlende Gelegenheiten für den Verstoß gegen Ernährungziele wie externe zeitliche oder örtliche Einflüsse (Arbeit, Studium oder andere Aktivitäten), die den Fokus vom Essen weg verschieben oder für die Einhaltung von Reduktionszielen sorgen.*

5.1. Beschäftigt, bei allgemeiner Beschäftigung oder wenn der Fokus auf einer bestimmten Beschäftigung liegt, z.B. „ich habe für die Uni gearbeitet“, „beschäftigt“, „Ablenkung“. „Lernen“, „Sport“, „Paintball“, „keine Zeit“, Zeitstress, „noch keine Gelegenheit Abend zu essen
5.2. Ortsbedingt / unterwegs, wenn der Fokus auf dem Ort liegt, z.B. „Uni“, „Arbeit“, „nicht daheim“, „Draußen essen“
5.3. Geschlafen, wenn Schlaf die Aufnahme von Nahrung verhindert hat, z.B. „bis jetzt geschlafen“, „Bin eingeschlafen und habe nichts gegessen
5.4. Andere Faktoren, z.B. „Zähne geputzt“, „morgens“

6. Verfügbarkeiten von Lebensmitteln

*Hier geht es um Faktoren, die die Zugänglichkeit und bewusste Auswahl gesunder beziehungsweise ungesunder Lebensmittel beeinflussen. Dabei umfasst die Kategorie die Zugänglichkeit zu bestimmten Lebensmitteln sowie die bewusste Entscheidung für Alternativen, um das Erreichen bestimmter Ernährungsziele zu unterstützen. Es geht vor allem um den eigenen Verantwortungsbereich der Personen, nicht um die Beteiligung Dritter.*

6.1. Fehlende Verfügbarkeit zu vermeidender Lebensmittel, z.B. „Nichts da“, „Keine Süßigkeiten greifbar“, „Keine Schokolade im Kühlschrank“, „Es gab kein Fleisch zu essen“
6.2. Einfache Verfügbarkeit von Alternativen, z.B. „Obst als Ersatz für Süßigkeiten“, „Gesunde Alternativen“, „vegetarische alternative“, „Leckere Alternative“, „Alternativen zur Verfügung“, „Gesunde Alternativen zuhause“

7. Soziale Faktoren und externe Motivation

*Umfassen Faktoren für die Einhaltung der Ernährungsziele, die auf dritte Personen oder Einflüsse zurückzuführen sind, z.B. externe Motivationsgründe.*

z.B. „Meine Freundin hat mich motiviert“, „Motivation durch diese Studie“, „Meine Mama hat was Proteinreiches gekocht“, „Freunde“, „Familie“, „Diese Erinnerung der App hat mich wieder an mein Ernährungsziel erinnert“, „Streit“

In ANDERES sind die Kategorien für nicht zuordenbare Aspekte/Segmente und Segmente, die z.B. keine Gründe für die Einhaltung/Nicht-Einhaltung von Ernährungszielen beinhalten, zusammengefasst. Bitte sparsam verwenden.

8. Keine Angabe von Gründen

Wenn keine Gründe angegeben wurden, *warum* ein Ernährungsziel eingehalten wurde. Zum Beispiel wird nur das Verhalten an sich beschrieben („keine Süßigkeiten gegessen“) aber kein Grund dafür.

9. Sonstiges

*Wenn das Segment einen Grund angibt, aber keiner vorhandenen Kategorie zugeordnet werden kann. Bitte sparsam verwenden.*

**Kategorienhandbuch für dietary lapses**

INTERNE Gründe für die Nicht-Einhaltung von Ernährungszielen haben unmittelbar mit dem eigenen Ernährungsverhalten einer Person zu tun. Dazu gehören:

1. Selbstregulation

Diese Kategorie umfasst die inneren Beweggründe, die dazu führen, dass Teilnehmende ihre Ernährungsziele nicht einhalten können, ob beabsichtigt oder unbeabsichtigt.

1.1. Fehlende Selbstkontrolle, z.B. „Schwache Impulskontrolle heute.“
1.2. Fehlende Motivation, z.B. „Unlust zu kochen“, „wenig Motivation ans Ziel zu denken“
1.3. Fehlende Planung, z.B. „keine vorbereitete Brotzeit“, „zu spät gegessen“, „zu wenig Mittag gegessen“, „Essen mit MHD für heute“
1.4. Geplante / bewusste Ausnahme, z.B. „ich tracke heute nicht weil wir Essen bestellen wollten und ich mal eine Pause vom Tracken brauche“, „Wochenende = 'Ausnahme', gekaufte Sachen von Eltern die weg sollten“, „hatte Müsli was ich ausprobieren wollte“, „Weil ich Nachtisch wollte“

2. Emotionale Faktoren

*Emotionale Zustände wie Frust, schlechte Laune, Langeweile, emotionaler Stress. Diese Faktoren stören die Zielerreichung durch emotionale Turbulenzen.*

2.1. Unangenehme Gefühle und Langeweile, z.B. „Schlechte Stimmung“, „Frust“, „Ängste“, „Langeweile“
2.2. Unterstützung zur Zustandsverbesserung, Essen als Stütze zur Verbesserung des emotionalen oder körperlichen Zustands, z.B. „Plünderung vom Süßigkeitenvorrat um ein gutes gefühl zu bekommen“, „Süßigkeiten zur Unterstützung zum Lernen, Energie“, „Motivations-Twix, um eine unangenehme Arbeit fertig zu stellen“, „Mit Süßem Belohnt“, „Energiegewinnung“, „hab Kaffee gebraucht“
2.3. Stress, als subjektives Empfinden [oder Zeitstress oder physiologische Reaktion bei fehlender Spezifizierung], z.B. „Stress“, „Ich hatte einen stressigen Tag“

3. Physiologische Faktoren

*Physische Zustände, die das Essverhalten beeinflussen.*

3.1. Gesundheitszustand, z.B. „[Müdigkeit], Unwohlsein“, „Schwindel“, „verkatert“, „PMS“, „Alkohol, hab getrunken“
3.2. Schlafbezogene Faktoren / Erschöpfung z.B. „Müdigkeit, [Unwohlsein]“, „körperliche Arbeit“, keine Energie
3.3. Hunger, Aspekte von Hunger und Sättigung (je nach Ziel), z.B. „Sehr hungrig“, „wenig Hungergefühl“, „Lange Zeit nichts gegessen“

4. Verlangen / Appetit

*Hier geht es um die Thematisierung von Verlangen, Lust, Appetit und Heißhunger oder die Abwesenheit dieser. Es kann um bestimmte Lebensmittel oder Essen generell gehen.*

z.B. „Lust auf Süßes“, „Weil alles so lecker war“, „Gusto“, „Ich hatte keine Lust auf etwas Proteinreiches“, „Heißhunger“, „Cravings“

EXTERNE Gründe für die Nicht-Einhaltung von Ernährungszielen haben nicht unmittelbar mit dem eigenen Ernährungsverhalten einer Person zu tun, sondern sorgen von außen für Versuchungen und Schwierigkeiten bei der Einhaltung der Ziele Dazu gehören:

5. Zeitmangel und Gelegenheiten

*Situationen durch Zeitmangel oder unplanbare Umstände, die das Einhalten der Ernährungsziele erschweren.*

z.B. „Keine Zeit zu kochen.“, „Viel gelernt, keine Zeit.“, „Hatte Uni und hab nix gegessen.“, „Keine Zeit, um gesunde Mahlzeiten zuzubereiten.“, „Unterwegs“, „war nicht zuhause“, Reise, Zugfahrt, „Musste was schnelles essen“, „Nichts gegessen“

6. Ungeplante Umstände

*Situationen, in die die Person hineinkommt, ohne sich selbst aktiv dafür entschieden zu haben, Situationen, die sich ereignen ohne Zutun, in denen die Person keinen Einfluss auf die Zutaten des Essens hat.*

*z.B. „heute war ein besonderer Tag“, „Spontanes Essengehen, keine Alternativen“,* „*ich hatte was von gestern übrig, das aufgegessen werden musste“, „Urlaub“*

7. Verfügbarkeiten von Lebensmitteln

*Zugänglichkeit, Verlockungen und spontane Versuchungen ohne aktive Beteiligung Dritter [🡪 soziale Faktoren] sowie die Präsenz offener Snacks, Süßigkeiten oder häufig gesehene Versuchungen.*

7.1. Einfache Verfügbarkeit zu vermeidender Lebensmittel, auch bloße Aufzählung von Lebensmitteln, z.B. „Schokolade war noch offen“, „Süßigkeiten im Büro.“, „Verfügbarkeit.“, „Essen war so verlockend.“, „Es gab Eis“, „Kuchen umsonst“, „Zucker“, „Kinder Bueno“
7.2. Fehlende Verfügbarkeit von Alternativen, z.B. „Keine Alternative vorhanden.“, „Verfügbarkeit ungesunder Lebensmittel.“, „Süßigkeiten zuhause gehabt.“, „Nahrungsmittel war in der Nähe.“

8. Soziale und gesellschaftliche Faktoren

*Einfluss sozialer Situationen (unter Beteiligung Dritter) wie Feiern und Treffen, aber auch gesellschaftlicher Druck verbunden mit Gruppenzwang oder Erwartungen, die das Verhalten beeinflussen.*

z.B. „Geburtstagsfeier von einer Freundin.“, „Mit Freunden zusammen.“, „Gesellschaftlicher Druck, wäre unhöflich gewesen.“, „Familienessen.“ „Es gab Donuts bei der Arbeit.“, „Hochzeit“, „Geselligkeit in der Runde“, „Dampfnudeln von Oma“, „[Es gab nichts anderes zu essen], ich wollte nicht nervig sein“, „*gekaufte Sachen von Eltern sollten weg“*,

In ANDERES sind die Kategorien für nicht zuordenbare Aspekte/Segmente und Segmente, die z.B. keine Gründe für die Einhaltung/Nicht-Einhaltung von Ernährungszielen beinhalten, zusammengefasst. Bitte sparsam verwenden.

9. Keine Angabe von Gründen

Wenn keine Gründe angegeben wurden, *warum* ein Ernährungsziel nicht eingehalten wurde. Zum Beispiel wird nur das Verhalten an sich beschrieben („habe Süßigkeiten gegessen“) aber kein Grund dafür oder es wird geschrieben „einfach so“, „war halt so“

10. Sonstiges

*Wenn das Segment einen Grund angibt, aber keiner vorhandenen Kategorie zugeordnet werden kann. Bitte sparsam verwenden.*
