# Supplementary material for: Nomothetic and idiographic determinants of dietary lapses and adherence: an exploratory mixed methods ecological momentary assessment study of open-text responses
Source: Front Nutr. 2026 Aug 14;13:1869531. doi: 10.3389/fnut.2026.1869531 (PMC13521853; doi:10.3389/fnut.2026.1869531)
Supplement: Supplementary file 1 [file Supplementary_file_1.zip › Supplement D.docx]

**Supplement D: Additional Tables**

**Table D1**

Raw frequencies and corresponding probabilities of reported lapse categories

| **Category** | | |  |
| --- | --- | --- | --- |
| **High** | **Mid-level** | **Lower-level** | **n (%)** |
| Internal | Lack of Self-regulation | No Self-control | 37 (3.31%) |
|  |  | No Motivation | 18 (1.61%) |
|  |  | No Planning | 9 (0.81%) |
|  |  | Deliberate* Exception | 57 (5.12%) |
|  |  | *Overall* | 121 (10.85%) |
|  | Emotional Factors | Negative Emotions* | 77 (6.90%) |
|  |  | Stress | 67 (6.00%) |
|  |  | Emotional Support | 15 (1.34%) |
|  |  | *Overall* | 153 (14.24%) |
|  | Physiological Factors | Health-related | 51 (4.57%) |
|  |  | Sleep-related | 42 (3.76%) |
|  |  | Hunger* | 99 (8.87%) |
|  |  | *Overall* | 186 (17.20%) |
|  | Cravings |  | 169 (15.14%) |
| *Overall* |  |  | 629 (56.98%) |
| External | (Un)availability | Goal-Incongruent Foods* | 100 (8.96%) |
|  |  | Alternatives | 39 (3.32%) |
|  |  | *Overall* | 139 (12.28%) |
|  | Opportunities |  | 148 (13.26%) |
|  | Social Factors |  | 144 (12.90%) |
|  | Unplanned Circumstances |  | 44 (3.94%) |
| *Overall* |  |  | 475 (43.03%) |

**Table D2**

Raw frequencies and corresponding probabilities of reported adherence categories

| **Category** | | |  |
| --- | --- | --- | --- |
| **High** | **Mid-level** | **Lower-level** | **n (%)** |
| Internal | Self-regulation | Self-control* | 436 (10.25%) |
|  |  | Motivation | 363 (8.53%) |
|  |  | Planning | 240 (5.64%) |
|  |  | Routines | 159 (3.74%) |
|  |  | Strategies | 135 (3.17%) |
|  |  | *Overall* | 1290 (31.33%) |
|  | Emotional Factors |  | 78 (1.83%) |
|  | Physiological Factors | Health-related | 58 (1.36%) |
|  |  | Sleep-related | 17 (0.40%) |
|  |  | Hunger* | 335 (7.87%) |
|  |  | *Overall* | 410 (9.63%) |
|  | No Craving |  | 506 (11.89) |
| External | (Un)availability | Goal-Incongruent Foods* | 282 (6.63%) |
|  |  | Alternatives | 188 (4.42%) |
|  |  | *Overall* | 139 (11.05%) |
|  | Lack of Opportunities | Occupied* | 616 (14.48%) |
|  |  | On the Way* | 431 (10.13%) |
|  |  | Sleeping | 146 (3.43%) |
|  |  | Other Factors | 156 (3.67%) |
|  |  | *Overall* | 1324 (31.71%) |
|  | Social Factors |  | 109 (2.56%) |

**Table D3**

*Indices for Mid-Level Categories combined for lapses and goal adherence*

| **Indices** | | | |  | | |  |  |
| --- | --- | --- | --- | --- | --- | --- | --- | --- |
| **High** | **Mid-level** | **n (%)** | **Average User Prevalence** | | **User Variance** | **Top-user probability** | | **User Coverage (≥1 occurrence**) |
| Internal | Self-regulation related factors | 1454 (27.07%) | 21.67% | | 40.38% | 58.93% | | 82.41% |
|  | Emotional Factors | 237 (4.41%) | 1.85% | | 42.06% | 7.46% | | 40.70% |
|  | Physiological Factors | 602 (11.21%) | 5.63% | | 33.58% | 19.66% | | 58.79% |
|  | Craving-related factors | 675 (12.57%) | 5.97% | | 39.07% | 27.30% | | 60.80% |
| External | Availability-related factors | 609 (11.34%) | 6.96% | | 37.49% | 27.33% | | 66.83% |
|  | Opportunity-related factors | 1497 (27.87%) | 23.57% | | 25.19% | 48.99% | | 81.41% |
|  | Social  Factors | 253 (4.71%) | 3.10% | | 24.01% | 7.97% | | 48.24% |
|  | Unplanned Circumstances | 44 (0.82%) | 0.33% | | 40.90% | 1.40% | | 16.58% |

**Table D4**
*Differences in Sample Characteristics between Study 1 and Study 2*

|  | Study | |  |  |
| --- | --- | --- | --- | --- |
|  | *Study 1*  *N = 149* | *Study 2*  *N = 50* |  |  |
| *Variable* | *M (SD)* | *M (SD)* | *X^2^/t (df)* | *p* |
| % female | *83.22%* | *83.67%* | 0.13 (2) | .939 |
| Age | *26.48* | *24.32* | 1.48 (68.66) | .144 |
| Body Mass Index | 24.60 | 22.45 | 2.74 (170.21) | .007 |

*Note.* For the proportion of female participants, we used a Person’s Chi-squared test and for differences in age and body mass index, the Welch t-test.
