# Supplementary material for: Nomothetic and idiographic determinants of dietary lapses and adherence: an exploratory mixed methods ecological momentary assessment study of open-text responses
Source: Front Nutr. 2026 Aug 14;13:1869531. doi: 10.3389/fnut.2026.1869531 (PMC13521853; doi:10.3389/fnut.2026.1869531)
Supplement: Supplementary file 1 [file Supplementary_file_1.zip › Supplement E.docx]

**Supplement E: Between-Person Heterogeneity**

**Figure E1**

*Predicted Probability per Mid-Level Lapse-Category and Participant*

**Figure E2**

*Predicted Probability per Mid-Level Adherence-Category and Participant*
