# Supplementary material for: Nomothetic and idiographic determinants of dietary lapses and adherence: an exploratory mixed methods ecological momentary assessment study of open-text responses
Source: Front Nutr. 2026 Aug 14;13:1869531. doi: 10.3389/fnut.2026.1869531 (PMC13521853; doi:10.3389/fnut.2026.1869531)
Supplement: Supplementary file 1 [file Supplementary_file_1.zip › Supplement F.docx]

**Supplement F: Sensitivity Analysis on User Coverage**

As User Coverage is inherently sensitive to the number of reported reasons contributed by each participant, we examined its sensitivity to reporting intensity by progressively excluding participants with few reported reasons for lapses. Specifically, we stepwise increased a cutoff (*j*) and retained only participants with more than *j* reported reasons, until only 20 participants remained. For each cutoff, User Coverage was recalculated as the proportion of remaining participants who reported each category at least once. This procedure was conducted separately for lapse and adherence categories. Due to the lower frequency of lapses, a minimum threshold of 13 reported reasons was required for 20 participants to remain. In contrast, for adherence, the corresponding threshold was 45 reported reasons.

As expected, User Coverage increased with stricter minimum reporting thresholds for both lapse and adherence categories. For lapses (Figure F1), all mid-level categories showed coverage below 50% when including all participants (i.e., cut-off of at least 1); however, coverage rose to as high as 80% for social and physiological factors when restricting the sample to the 20 participants with at least 13 reported lapse reasons. A similar pattern emerged for goal adherence categories (Figure F2). User Coverage increased to over 90% (92.59%) for self-regulation-related factors and lack of opportunities when limiting the sample to the 20 participants with at least 45 reported success reasons, compared to a maximum of 80.61% when all participants were included using a cut-off of at least 1 reported reason. Figures F3 and F4 show equivalent plots but for the lower-level lapse and adherence categories. For lapses, the highest User Coverage was observed for hunger (55%), while all other categories remained below 50%, even when restricting analyses to participants with the strongest data basis. For adherence, the highest User Coverage reached 88.88% for being occupied and 80% for being on the way. However, half of the lower-level categories (i.e., seven) remained below 50%, even when focusing on participants with the strongest data basis.

**Figure F1**

*User Coverage under increasing minimum-reporting cutoffs per participant for mid-level lapse categories*

**Figure F2**

*User Coverage under increasing minimum-reporting cutoffs per participant for mid-level adherence categories*

**Figure F3**

*User Coverage under increasing minimum-reporting cutoffs per participant for lower-level lapse categories*

**Figure F4**

*User Coverage under increasing minimum-reporting cutoffs per participant for lower-level adherence categories*
